# “Mean ± SEM” or “Mean (SD)”?

**DOI:** 10.4103/0253-7613.70402

**Published:** 2010-10

**Authors:** Jaykaran

**Affiliations:** Department of Pharmacology, Government Medical College, Surat, India. E-mail: drjaykaran@yahoo.co.in

Sir,

Use of descriptive statistics is very common in articles published in various medical journals. For the ratio and interval data following the normal distribution, the most common descriptive statistics is mean and standard deviation (SD) and for data not following the normal distribution, it is median and range. It is, however, observed in various medical journals that mean and standard error of mean (SEM) are used to describe the variability within the sample.[1] We, therefore, need to understand the difference between SEM and SD.

The SEM is a measure of precision for an estimated population mean. SD is a measure of data variability around mean of a sample of population. Unlike SD, SEM is not a descriptive statistics and should not be used as such. However, many authors incorrectly use the SEM as a descriptive statistics to summarize the variability in their data because it is less than the SD, implying incorrectly that their measurements are more precise. The SEM is correctly used only to indicate the precision of estimated mean of population. Even then however, a 95% confidence interval should be preferred.[1,2] Further, while reporting mean and SD, instead of writing “mean ± SD” the better way of representation would be “mean (SD)” as it will decrease the chance of confusion with confidence interval.[2]
